# Autoimmune myocarditis associated with subcutaneous panniculitis-like T-cell lymphoma without HAVCR2 germline mutation

**DOI:** 10.1093/ehjcr/ytag610

**Published:** 2026-08-19

**Authors:** Rui Katano, Souichi Shiratori, Satonori Tsuneta, Atsushi Tada

**Affiliations:** Department of Cardiovascular Medicine, Faculty of Medicine and Graduate School of Medicine, Hokkaido University, Kita 15, Nishi 7, Kita-ku, Sapporo, Hokkaido 060-8638, Japan; Department of Hematology, Hokkaido University Faculty of Medicine, Kita 15, Nishi 7, Kita-ku, Sapporo, Hokkaido 060-8638, Japan; Department of Radiology, Graduate School of Dental Medicine, Hokkaido University, Kita 13, Nishi 7, Kita-ku, Sapporo, Hokkaido 060-8586, Japan; Department of Cardiovascular Medicine, Faculty of Medicine and Graduate School of Medicine, Hokkaido University, Kita 15, Nishi 7, Kita-ku, Sapporo, Hokkaido 060-8638, Japan

**Keywords:** Myocarditis, Subcutaneous panniculitis-like T-cell lymphoma, Multimodality

## Summary

A 22-year-old man with subcutaneous panniculitis-like T-cell lymphoma presented with cardiac dysfunction. Multimodality imaging suggested autoimmune myocarditis, despite the absence of HAVCR2 mutations. The patient improved with prednisolone, highlighting the importance of considering autoimmune myocarditis in SPTCL cases.

## Case description

**Figure 1:**
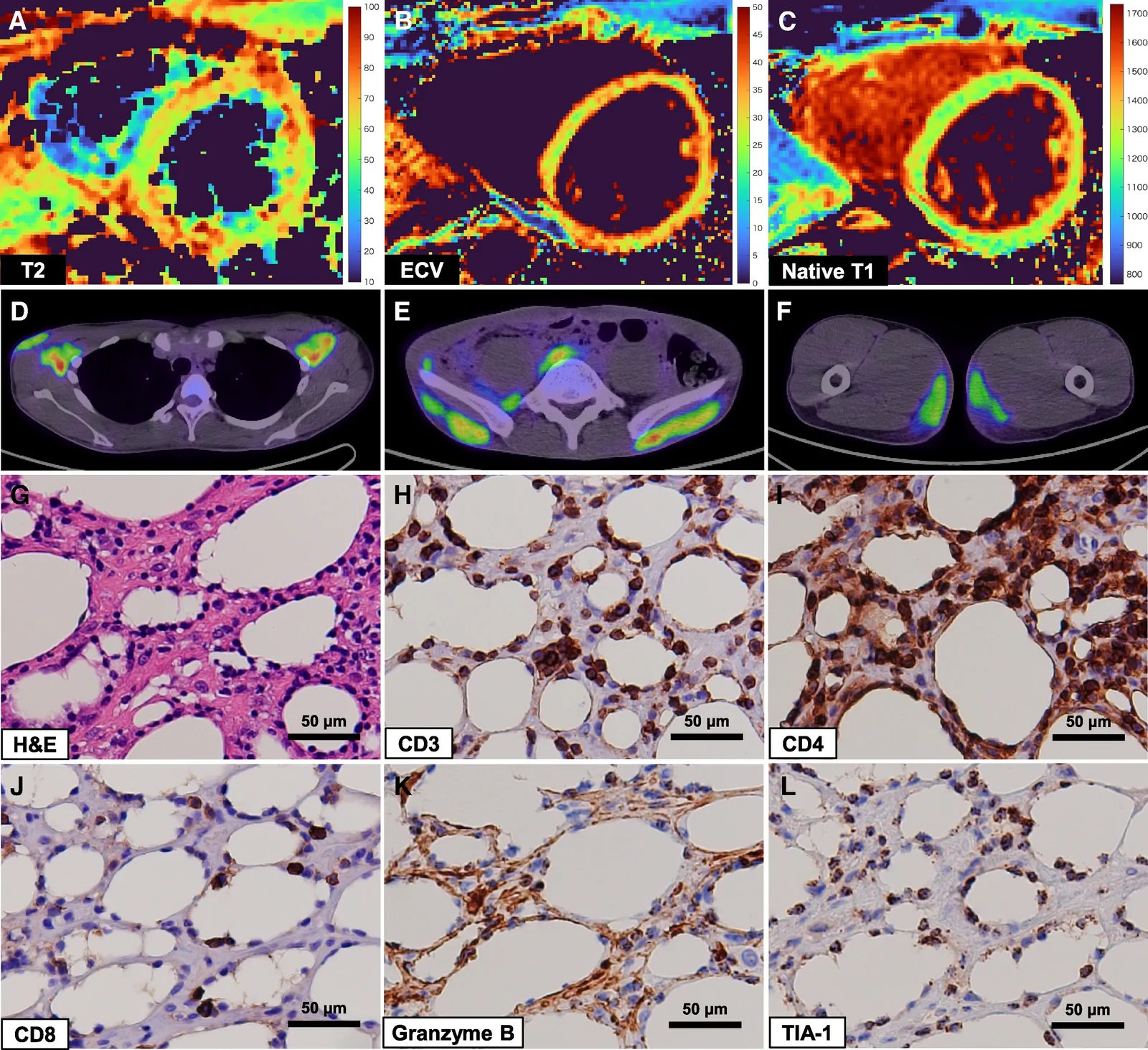
Cardiac magnetic resonance imaging showed increased T2 values (Panel *A*) and mildly elevated extracellular volume (Panel *B*), with normal native T1 values (Panel *C*), suggesting myocardial inflammation without fibrosis. ^18^F-FDG PET demonstrated intense uptake in subcutaneous adipose tissue of the axilla, lumbogluteal region, and inner thighs (Panel *D–F*) without myocardial uptake. Axillary biopsy showed atypical lymphocytic infiltration in the subcutaneous fat with rimming of adipocytes (Panel *G*). Immunohistochemical staining was positive for CD3, CD4, CD8, granzyme B, and TIA-1 (Panel *H–L*).

A 22-year-old man presented with fever and chest pain. Laboratory testing revealed pancytopenia with markedly elevated soluble interleukin-2 receptor and ferritin levels, whereas cardiac biomarkers were normal. Transthoracic echocardiography demonstrated diffuse biventricular hypokinesis with a reduced left ventricular ejection fraction of 40%, without pericardial effusion or apparent myocardial wall oedema (see Supplementary material online, *Videos S1–S3*). Coronary computed tomography angiography ruled out stenosis. Cardiac magnetic resonance imaging revealed increased T2 values (Panel *A*) and mildly elevated extracellular volume fraction (Panel *B*), with normal native T1 values (Panel *C*), suggesting myocardial inflammation without fibrosis (*Figure 1*). ^18^F-FDG PET demonstrated intense uptake in subcutaneous adipose tissue of the axilla, lumbogluteal region, and inner thighs (Panel *D* and *F*) without abnormal myocardial uptake. Axillary biopsy showed atypical lymphocytic infiltration in the subcutaneous fat with rimming of adipocytes (Panel *G*). Immunohistochemical staining was positive for CD3, CD4, CD8, granzyme B, and TIA-1 (Panel *H–L*), and negative for CD20, CD30, CD68, and EBER-ISH. Endomyocardial and bone marrow biopsies showed non-specific findings. PCR analysis detected monoclonal TCR-β gene rearrangement; germline HAVCR2 mutation was absent. This patient was diagnosed with subcutaneous panniculitis-like T-cell lymphoma (SPTCL) and treated with prednisolone and cardioprotective medications.

SPTCL is a rare subtype of peripheral T-cell lymphoma affecting younger patients. According to the previous studies, SPTCL is associated with autoimmune and infectious diseases.^1^ A previous report has documented autoimmune myocarditis associated with HAVCR2 germline mutations,^2^ which are frequently identified in patients with SPTCL. Although HAVCR2 mutation was absent in this case, the coexistence of cardiac dysfunction with systemic immune activation suggests autoimmune myocarditis sharing a common immunopathological mechanism with SPTCL.

This case highlights the importance of considering autoimmune myocarditis in patients with cardiac dysfunction and suspected SPTCL, warranting multimodality evaluation.

## Supplementary Material

ytag610_Supplementary_Data

## Data Availability

No new data were generated or analysed in support of this research.

## Notes

**Consent:** Informed consent was provided by the patient.
